# 955. Catching Up with the Times: Improving Patient Outcomes and Staff Satisfaction with Electronic Documentation

**DOI:** 10.1093/jbcr/irag033.522

**Published:** 2026-04-06

**Authors:** Morgan McNeely, Christianna Fabiano, Michaela M Craig, Sendra Starnes, James H Holmes, IV

**Affiliations:** Atrium Health Wake Forest Baptist, Winston-Salem, NC; Atrium Health Wake Forest Baptist, Winston-Salem, NC; Atrium Health Wake Forest Baptist Burn Center, Winston-Salem, NC; Atrium Health Wake Forest Baptist, Winston-Salem, NC; Atrium Health Wake Forest Baptist Burn Center, Winston-Salem, NC

## Abstract

**Introduction:**

Weekly multidisciplinary rounds (MDR) are essential to providing quality care to burn patients. These meetings encourage collaboration amongst team members, allow all to voice patient specific concerns, and to receive real-time feedback to navigate any issues so quality care can be provided to all patients. Historically our institution documented these meetings on paper, then scanned the forms into the patient charts. These forms were often left blank or partially filled out as they had not been updated parallel to electronic medical records (EMR), thus they were outdated or not relevant to each discipline. This performance improvement project aims to increase collaboration, communication and relevance of the topics discussed during MDR, while directly entering the documentation into the EMR.

**Methods:**

In December 2024, surveys were disseminated to each teammate involved in MDR. These gathered data from each discipline prior to making any changes; to discover what each felt was relevant to the entire team, including aspects of care to highlight in this weekly meeting. When reviewing the results, many felt paper charting was “outdated,” while others did not find the information relevant to their discipline, or their discipline was not included at all. In January 2025, a “smartphrase” (pre-formatted note) was created to document MDR in real-time in the EMR and went live in February 2025.

Every six weeks after go-live, another survey was conducted to improve the “smartphrase.” Survey feedback resulted in content items being added / removed to each discipline, and format changes being made, including the creation of a separate pediatric “smartphrase.”

**Results:**

From February 3, 2025, to April 24, 2025, all burn patients were documented on utilizing the “smartphrase” at least once. Survey feedback shifted the EMR documentation of the “smartphrase” from the physician assistants to the charge nurse and wound care nurse. This improved time management and provided more accurate nursing insight during MDR, which was a positive process change.

While some feel the new process is “more time consuming,” others stated they feel each discipline is “heard” and able to “voice their concerns with no interruptions.” Many stated it is a “good systematic checklist to ensure proper standard of care” and it helps with developing a “good plan of care” and they were “less likely to miss valuable information.”

**Conclusions:**

Overall, the new MDR template has been well received with positive feedback from most stakeholders involved. This project will be ongoing for continued improvement, with the hopes of maintaining this process as the new standard for the weekly MDR.

**Applicability of Research to Practice:**

This process change continues to meet all requirements for the ABA standards and will allow for better tracking of MDR for future reverification processes. It also highlights the need to continue to modernize practices to match the current expectation of documentation within the EMR.

**Funding for the study:**

N/A.

